# A Fuzzy Model of Risk Assessment for Environmental Start-Up Projects in the Air Transport Sector

**DOI:** 10.3390/ijerph16193573

**Published:** 2019-09-24

**Authors:** Volodymyr Polishchuk, Miroslav Kelemen, Beáta Gavurová, Costas Varotsos, Rudolf Andoga, Martin Gera, John Christodoulakis, Radovan Soušek, Jaroslaw Kozuba, Jakub Hospodka, Peter Blišťan, Stanislav Szabo

**Affiliations:** 1 Faculty of Information Technologies, Uzhhorod National University, 88000 Uzhhorod, Ukraine; 2 Faculty of Aeronautics, Technical University of Kosice, 04121 Kosice, Slovak Republic; miroslav.kelemen@tuke.sk (M.K.); rudolf.andoga@tuke.sk (R.A.);; 3 Research and Innovation Centre Bioinformatics, USP TECHNICOM, Technical University of Košice, 040 01 Kosice, Slovak Republic; beata.gavurova@tuke.sk; 4 Department of Physics, National & Kapodistrian University of Athens, GR-15784 Athens, Greece; covar@phys.uoa.gr (C.V.); ichristo@phys.uoa.gr (J.C.); 5 Faculty of Mathematics, Physics and Informatics, Comenius University in Bratislava, Bratislava, 84248 Mlynska Dolina, Slovak Republic; mgera@fmph.uniba.sk; 6 Faculty of Transport Engineering, University of Pardubice, 53210 Pardubice, Czech Republic; radovan.sousek@upce.cz; 7 Faculty of Transport, Silesian University of Technology, 44100 Gliwice, Poland; jaroslaw.kozuba@polsl.pl; 8 Faculty of Transport, Czech Technical University in Praque, 16000 Praque, Czech Republic; xhospodka@fd.cvut.cz; 9 Faculty of Mining, Ecology, Process Control and Geotechnology of Aeronautics, Technical University of Kosice, 04121 Kosice, Slovak Republic; peter.blistan@tuke.sk

**Keywords:** air transport sector, decision support systems, environmental start-up project, expert systems, expert evaluation, fuzzy model, financial investments, decision-maker (DM), risk assessment

## Abstract

The purpose of this paper is to develop a fuzzy model of the risk assessment for environmental start-up projects in the air transport sector at the stage of business expansion. The model developed for the following software will be a useful tool for the risk decision support system of investment funds in financing environmental start-up projects at the stage of market conquest. Developing a quantitative risk assessment for environmental start-up projects for the air transport sector will increase the resilience of making risk decisions about their financing by the investors. In this paper, a set of 21 criteria for assessing the risk of launching environmental start-up projects in the air transport sector were formulated for the first time by presenting inputs in the form of a linguistic risk assessment and the number of credible expert considerations. The fuzzy risk assessment model, based on expert knowledge, uses linguistic variables, reveals the uncertainty of the input data, and displays a risk assessment with linguistic interpretation. The result of the paper is a fuzzy model that is embedded in a generalized algorithm and tested in an example risk assessment of environmental start-up projects in the air transport sector.

## 1. Introduction

In today’s globalized economy, transport is tasked with overcoming longer distances, while becoming more reliable and environmentally friendly. The transport sectors must do their utmost to minimize transport costs [1] and to develop green/environmentally friendly transport [2,3]. In recent years, there was a tendency to increase the number of air flights, which has a negative impact on environmental pollution. At the same time, attention is paid to the need for environmentally friendly transportation solutions and optimization of carbon dioxide emissions [1,2]. An environmental start-up project in air transport is a project based on innovative technologies that aims to reduce the negative impact of the sphere of air transport on the environment, or to address specific eco issues.

At present, there is demand in the field of air transport for green environmental transport start-up projects. This is evidenced by a large number of environmental start-ups improving the environmental performance of airlines and airports, for services operating in the air transport industry, improving air traffic management, creating new environmental means for air transport, etc. At the same time, there is a need to finance such projects for their introduction into the market and in the future to conquer the market. Financing environmental start-ups for air transport systems is risky. The number of companies operating in the aviation sector which launch their own venture capital or crowdfunding platforms is steadily increasing [4]. There are various options for financing start-up projects in the air transport sector, but they all have one issue: finding and financing a successful project with minimal risks. Appropriate risk-based decision support systems and risk management policies must be in place to minimize risks.

The regional and global competitiveness of economies are related very closely linked to innovative activities. Start-ups are among small and medium-sized enterprises (SME), with a share of up to 99.9% of all business entities in the Slovak Republic. The pan-European trend marks a steady decline in self-employment preferences over employment, and since 2009 this figure fell from 45% of Europeans doing business to 37% (it is only 33% in Slovakia). These figures are higher in some business-oriented countries, such as the United States of America (USA) (51%) and China (56%) [5]. Compared to other European Union (EU) countries, Slovakia has the highest share of micro-enterprises. From a structural point of view, small and medium-sized enterprises in Slovakia have significantly higher representation in the industrial and construction sectors than in the EU-28, as opposed to services [6]. In terms of economic indicators, the effectiveness of small and medium-sized companies in Slovakia is relatively low compared to other countries [7]. It is affected not only by the structure and size of companies, but also by the stated insufficient level of expenditure on science and research, the low level of productivity of companies, and various other factors, as outlined in References [8,9,10]. This is consistent with the long-term orientation of small and medium-sized companies in Slovakia, in particular with regard to technology imports, and it indicates the inadequacy and lack of support schemes for their development and impact [11,12]. These have a significant impact on the lifetime of start-up companies, with the Slovak Republic taking the penultimate position in EU countries in this respect. Only 41.7% of newly created SMEs survive after three years, with the EU average of 56.1% [6]. On a national scale, we are seeing a slight improvement, as evidenced by data on the number of business start-ups/deaths in 2017. The number of small and medium-sized enterprises created increased by 11.2% from an annual perspective, and the number of business deaths increased by 2.4% [6].

One of the ways to develop innovative SMEs that provide sufficient competitive strength is to support start-ups. The available resources indicate that there are about 600 start-ups in Slovakia with more than 3000 employees. Statistics show that up to 85% of Slovak start-ups are in their early stages of development, with 41% in the so-called beta phase; 50% of them generate revenue, but 83% of start-ups are under €100,000. The main sources of start-ups funding are savings (74%) and support from families and friends (22%). Some start-ups (57%) are considering relocating to other countries, focusing not only on new markets and customers (80%), but also on access to finance (48%), as well as tax and legislative environment (32%) [13].

These statistics create a coherent picture of the state, development, and determinants of the growth of start-ups in Slovakia. Thus, attention is drawn to the need to develop optimal stabilization and regulatory mechanisms, with platforms applied to prepare various support initiatives. This long-term process must be systemic and interactive. The internal environment of start-up development (university science parks, research institutions, etc.) should be some of the initiators of changes related to activation of support schemes, better support for cooperation within the start-up system, incentives for business activities, etc. The lack of feedback from the business environment or underestimating the clear negative growth trends can have a fatal impact on innovation and start-up development in the future. The following characteristics, which are critical for the development of start-ups in Slovakia, also raise the need for systemic solutions [14]:

1. Long-term shortage of financial and non-financial instruments.
2. Inadequate interconnection of start-up communities with scientific and research institutions, or universities.
3. Excessive legislative burden on business.
4. Inadequate cooperation between individual entities of the start-up ecosystem.
5. Poor entrepreneurship motivation, coupled with a lack of business skills.

These defined characteristics are of a considerable macroeconomic nature, but their quantifiable effects on the microeconomic framework for start-up development are evident in the statistics above. Macroeconomic characteristics are subject to change very slowly, depending on state policies, as well as the potential of its economy, social, and economic development. On the other hand, it is possible to change the economic outcome indicators if knowledge of the internal environment of start-ups development dominates, and the environment is studied in detail. This is the role of multiple entities in a start-up ecosystem, which can correctly identify analytic structures through expert processes.

The role of new methodologies and models, in the real data being designed, and the evaluation of the impact of critical factors on the emergence and development of start-ups come to attention. Of particular importance are the methodologies and models that quantify the level of risk at various stages of start-up development, with a dynamic structure reflecting changes in their external (macroeconomic) and internal (microeconomic) environment. They can also be an active structure in setting up monitoring and evaluation mechanisms and supporting the results of the internal strategies of university centers and their interconnection with other entities in the start-up ecosystem.

Today’s consolidated methodologies for assessing the risks of companies or projects are coherent on the basis of common economic parameters and quantitative indicators, which do not capture the so-called soft factors. This greatly limits their dissemination dimension and their applicability to practice and to different types of policies. Strategic frameworks, development concepts or various programs, and support for business development and support for start-up must be based mainly on feedback from the real environment of start-ups functioning, and this does not exist in Slovakia (among other places).

The implementation of quality analyses revealing the critical points of start-up development will also test the adaptability of several methods to explore specific conditions for the development of start-ups either in sectors or developmentally (depending on the stages of the company, start-up development), differentiating and further supporting methodological developments, as well as national and international benchmarks. This is extremely important for the creation of a global start-up system, which is also of interest to several international documents.

Innovation, as compared to other activities, is more risk-related, since there is virtually no full guarantee of a positive result. As a result, environmental start-up air transport projects are more dependent on the uncertainty factors that cause the risks. Risks are generated by ignorance regarding the future of start-up projects and limited views on an existing problem. Decisions made in the face of limited knowledge may lead to mistakes in the future. Moreover, current risk assessment methods do not take into account subjectivism, leading to incorrect estimates. False risk assessments lead to funding losses.

In response to these facts, it was decided to carry out this research, with the main aim of developing a fuzzy model of risk assessment for environmental start-up projects in the air transport sector at the stage of business expansion. The model developed for the following software will be a useful tool to support decision-making processes, especially in initial business initiatives where most start-ups fail. The results of our study will also support the further development of methodologies for assessing this economic sector, taking into account their high degree of expertise and innovation, including sectoral differentiation aspects and life cycle.

Therefore, the purpose of the work was to develop a fuzzy model of risk assessment for environmental start-up projects in the air transport sector in the stage of business expansion (avalanche-conquering market), as investment companies in the aviation industry are interested in the solution. 

The main motive for research into this issue and its contribution was an expert model for creating an software (SW) tool for practical use of risk assessment of start-up projects with solutions in the field of environmental projects in aviation (green transport) to strengthen the environmental and public health protection, at the University Science Park TECHNICOM ecosystem in Kosice and the start-up incubator at the Uzhhorod National University. Subsequently, this SW tool will be provided with the authors’ knowledge to our project partners for academic purposes, to protect investments and improve the following start-up projects. 

What is the motivation for the transport sectors to try their best to develop green/environmentally friendly transport? In 2014, United Nations Secretary-General Ban Kimoon appointed an independent High-Level Advisory Group on Sustainable Transport to provide a focused set of recommendations on how the transport sector can advance sustainable development with poverty eradication at its core, promote economic growth, and bolster the fight against climate change. The High-Level Advisory Group defines sustainable transport as the provision of services and infrastructure for the mobility of people and goods—advancing economic and social development to benefit today’s and future generations—in a manner that is safe, affordable, accessible, efficient, and resilient, while minimizing carbon and other emissions and environmental impacts (see the Mobilizing Sustainable Transport for Development: Analysis and Policy Recommendations from the United Nations Secretary-General’s High-Level Advisory Group on Sustainable Transport). Unfortunately, air transport, as part of the transport sector, contributes to the negative impacts on the environment and public health. For this reason, the social demand for creating eco-friendly aviation projects is growing.

The demand of the transport sector for start-up environmental projects is mainly due to the diversity of solutions, as well as modern, flexible, and often unconventional approaches to accelerate the implementation of environmentally friendly transport, in order to eliminate the serious environmental and public health impacts generated by traffic. Start-up incubators can be an important source of these projects. The environmental project means a time- and subject-limited set of tasks that are implemented in an interdisciplinary manner, focusing on environmental protection with an impact on public health. Environmental projects for aviation bring solutions to the practical problems related to eco-friendly airline management, green aviation technology, and innovation. The risk assessment in such projects must be more stringent in the light of the specific activity in the aviation sector with passenger and cargo transport, taking into account the risks of implementing environmental measures and their effects. Last but not least, there are risks associated with the possible fraudulent conduct of environmental start-up project-makers at the crime level.

## 2. Literature Review

Start-ups have different stages of commercial development. The first stage is the output of the product on the market. This raises the problem of the concept of evaluation itself, as the data for a non-implemented project are expert and fuzzy [15,16]. For this task, the authors already conducted research on the topic “fuzzy model for quantitative assessment of the environmental start-up projects in the air transport”. After the successful completion of the first stage, the second will come—conquering the market as a competitive factor in the industry. To assess the financing of the second stage, there are large number of developed models, but they are identified by the evaluation of investment projects and mainly used quantitative approaches [17,18]. In addition, if you consider the nature of the start-up projects, then the stage of new financing (expansion) may come in a few months after the successful launch. As a result, the quantitative indicators of the firm’s activities are not sufficient to apply classical methods of evaluating investment projects. In addition, in order to assess the feasibility of financing start-up projects, it is necessary to employ expert knowledge on the risks of such financing.

The issue of quantifying risk during an investment was presented in many papers [19,20,21], but a holistic concept of determining the level of risk in the subjective aspects of evaluation is yet to be developed. There are a number of works that offered project risks assessment using the net present value (NPV) formula [22]. In papers such as References [23,24], fuzzy sets, fuzzy logic, and a systematic approach to project risk assessment were used. In addition, in papers such as References [4,25,26], the authors proposed a formal model for project risk assessment, but did not address the question of risk assessment of environmental start-up projects for the air transport sector at the stage of business expansion. 

The main advantage of using a fuzzy set is that it requires a person who decides how to compare non-point probabilistic estimates, but at an interval, and this shows the corridor of the values of the predicted parameters. The convenience of these methods is manifested in increasing the degree of validity of decisions, as all possible scenarios of development depicting the continuous spectrum of the set of scenarios are taken into account [27,28,29]. Other experts and authors as in References [30,31] discussed the general ideas and advantages that underpin contemporary views on the use of fuzzy logic in decision support systems in various fields of application. To competently assess the risk of a start-up project, one must learn to scientifically model information uncertainty by drawing the formal boundaries between credible knowledge, knowledge with a certain level of certainty, and what we do not know. We also find inspiration in the work of authors on the “combination of data-driven active disturbance rejection and Takagi–Sugeno fuzzy control with experimental validation on tower crane systems” [31], or in the work on “density peaks clustering based on k-nearest neighbors and principal component analysis” [28], among others. We take advantage of experience from aviation risk assessment processes, as well as from work such as on the “security management education and training of critical infrastructure sectors’ experts” focused on transportation [32], on the model of supplier quality management in transport companies [33], or on the implementation of free route airspace (FRA) [34], among others.

Risk is closely linked to the notion of economic security of the project, both as the security of the entity representing the project and the safety of the investor [4]. The security of the matter is that a risky and unsuccessful project will result in enterprise damage. Investor safety depends directly on an adequate assessment of the project and the subject of the project. Enhancing the security of start-up projects provides stability of the economy of the region/state/EU [26]. In general, the problem of evaluating start-ups can be formalized as a decision-making problem, which is commonly solved using various formalized methodologies such as multi-criteria decision-making, expert systems, fuzzy inference systems, or their combinations [35,36]. All these methodologies, mentioned in the last paper, rely on the transfer of expert knowledge to a complex set of rules. However, transferring expert knowledge is a heuristic process [37]. On the other hand, the mechanism of neural network training is not based on human expertise; however, through a homogeneous structure of neural networks [38], structured knowledge is difficult to extract. The selected theoretical framework was also part of References [39,40] on the applied knowledge of interdisciplinary investigation of special security issues.

The plan was to compare at least three relevant methods for the objective and comprehensive solution of environmental start-up projects in the air transport sector. The first part of the study focuses on innovative solutions used by neuro-fuzzy systems for quantitative project evaluation and risk assessment. This paper presents the risk assessment in the first part of the ongoing study. The second part of the study uses one of the multi-criteria decision-making methods [35]. The third part of the study uses a selected expert system [35,37]. The knowledge gained in individual research questions enables the relevant methods for these purposes to be compared and to formulate the practical conclusions drawn. The authors intend to use these conclusions for a complex evaluation of environmental start-up projects in the University Science Park TECHNICOM ecosystem in Kosice and the start-up incubator at the Uzhhorod National University.

Thus, developing a fuzzy model for risk assessment of environmental start-up projects in the air transport sector is an urgent task in developing decision support systems for business analysts in assessing business financing opportunities. 

## 3. Materials and Methods 

### 3.1. Definition of the Assessment Problem

Modeling uses expertly generated information that reflects the substantive features of the object under study, and it is formulated in a natural language. In this case, the description of the object is unclear. Therefore, it is advisable to use fuzzy set theory to reflect object knowledge. Thus, the transition of knowledge in the classical sense proceeds to the fuzzy knowledge. To do this, fuzzy multiple descriptions are used to model uncertainty [27,28,29,30,31].

The configuration of the evaluation problem involved multiple steps. Firstly, the environmental start-up projects in air transport $S_{1},S_{2},\ldots ,S_{n}$ are considered, for which it is necessary to assess the risk of financing them at the expansion stage. Start-up projects are evaluated on the basis expert input estimates proposed by a set of criteria, $K=(K_{g1},K_{g2},\ldots ,K_{gm})$, which is classified in groups g. Experts evaluate each risk criterion [4] under one of the conditions of the following definition of linguistic variables $T=\left\{\begin{array}{ccccc} L; & BA; & A; & AA; & H \end{array}\right\}$, where L is “low risk”, BA is “risk below average”, A is “average risk”, AA is “risk above average”, and H is “high risk”.

For each risk assessment, the expert quantifies the “certainty” μ(T) [4,26] of his/her consideration in the interval [0, 1]. The input data for assessing the risk of environmental start-up projects in the air transport sector are presented in Table 1.

In Table 1, $T_{gij}$ is a variable with the T term defined for the *i*-th group indicator g and the *j*-th start-up project, and $\mu (T_{gij})$ is the accuracy of the expert estimates related to the suitability of the variable $T_{gij}$, where $i=\bar{1,m}$, $j=\bar{1,n}.$

Consequently, on the basis of the inputs submitted for environmental start-up projects in the field of air transport, it is necessary to assess their financing risk.

### 3.2. Knowledge Models for Risk Assessment of Environmental Start-Up Projects in the Air Transport Sector

We offer a set of criteria for forecasting the potential risks of environmental start-up projects in the air transport sector. A set of criteria were set up to predict the potential risks of environmental projects in the aviation sector, resulting from the consensus of several experts from the start-up ecosystems. An expert meeting was held with selected entities of emerging start-up ecosystems (investors, co-working sites, state organizations, start-up support organizations, corporations, and start-ups). There were also a set of criteria based on the expert evaluation of our researchers and colleagues from the University Science Park TECHNICOM ecosystem in Kosice and the start-up incubator at the Uzhhorod National University. This issue will be the subject of ta later paper, which will also be part of a research project supported by SAIA (Slovak Academic Information Agency in Bratislava, the Slovak Republic).

In our view, the proposed classification can be applied to all start-up projects. On the other hand, the peculiarities of the environmental start-ups of air transport projects are taken into account, as firms aim to create a product that solves current problems, by identifying markets, and scaling up business rapidly. The classification offers the following groups: KC—“risks from the current start-up project of the company“; KM—“risks of motivating a start-up team“; KI—“risks of initial investment and business models“; KF—“risks of a financial activity“; KS—“risks for developing an environmental start-up for air transport sector projects“.

A set of indicators represent each group of criteria [23,26]. The criteria proposed for the evaluation process by their description (groups) are defined and summarized in Table 2.

The set of risk criteria cannot reveal all aspects of a company’s activity in any environmental launch of air transport projects in different sectors of activities. Therefore, the set is open. Decision-makers (DMs) and the group of other ecosystem experts who know how they can contribute to the development of a set of relevant criteria that change over time (depending on changes in the external and internal environments) can always add additional risk criteria. The model is built in a way that does not depend on the number of criteria in the group. This allows the expert more flexibly, based on his/her experience, to add criteria based on knowledge of the real project.

### 3.3. The Fuzzy Mathematical Model for Quantitative and Linguistic Risk Assessments for Environmental Start-Up Air Transport Projects

A mathematical model for risk assessment for environmental start-up projects in the air transport sector is described, based on linguistic input variables. In the first stage, it is necessary to establish the membership rules and the knowledge base in order to reach the resulting term-evaluation $T_{g}$ for each group of risk criteria, and to determine the aggregated estimation of certainty $\mu (T_{g})$. In the second stage, based on the estimates obtained $T_{g}$ and $\mu (T_{g})$, we define a project risk assessment for each group of criteria g [26].

Consider the first stage—the construction of the membership rules that result from the term-evaluation of risk criteria groups.

Analyze an object from m inputs and the following output:

(1) $$T_{gj}=L(T_{g1j},T_{g2j},\ldots ,T_{gmj}),$$

where $T_{gj}$ is the resulting term-evaluation with a term-set T for a group of criteria g, and $T_{g1j},T_{g2j},\ldots ,T_{gmj}$, and are the input linguistic evaluation criteria for the group g. L is the operator that matches the resulting term-evaluation $T_{gj}$ for a group of criteria, for input variables $T_{g1j},T_{g2j},\ldots ,T_{gmj}$ (rule of logical output), where $j=\bar{1,n}$ [41,42].

Next, an expert (or a group of experts) builds the rules of membership of the resulting terms for everyone. These rules can be constructed as a percentage of the membership of one or another term of the input variable. Formally, the rules of a membership represent a system of logical utterances, “if, then, else” [43], which associate the values of the input variables $T_{g1j},T_{g2j},\ldots ,T_{gmj}$ with one of the possible values $T_{gj}$, g={O;M;I;F;S}, $j=\bar{1,n}$, as shown below.

If ($K_{g1j}=L$ and $K_{g2j}=L$ and … and $K_{gmj}=L$) Or ($K_{g1j}=L$ and $K_{g2j}=L$ and … and $K_{gmj}=BA$) Or … Or ($K_{g1j}=BA$ and $K_{g2j}=L$ and … and $K_{gmj}=L$) Then $T_{gj}=L,$ Else …

Similarly, all functional dependencies are formed, which embodies the rules of decision-making reduced to the knowledge base in mathematical form. 

The following rules of membership were formulated as a result of practical experience in the risk assessment of start-up projects, as done in References [4,25,26]:

**Level L**—“low risk”: the environmental start-up air transport project receives the resulting term-evaluation L, if the minimum number of criteria with the term “low risk” is not less than 60%, and the remaining 40% of the criteria at the level are not lower than that of “risk below average”.

**Level BA**—“risk below average”: the environmental start-up air transport project receives the resulting term-evaluation BA, if the minimum number of criteria under “risk below average” is at least 60%, with the remaining 40% at a level not lower than the “average risk”.

**Level A**—“average risk”: the environmental start-up air transport project receives the resulting term-evaluation A, if the minimum number of criteria with the term “average risk” is at least 60%, and the remaining 40% level is not lower than “risk above average”.

**Level AA**—“risk above average”: the environmental start-up air transport project receives the resulting term-evaluation AA, if the minimum number of criteria with the term “risk above average“ is at least 60%, and the remaining 40% of the criteria can be deemed “high risk”.

**Level H**—“high risk”: the environmental start-up project in air transport receives the resulting term-evaluation H, if the minimum number of criteria with the term “high risk” is 60% or more. 

Then, based on the rules for membership in the resulting term, the evaluation of risk criteria groups, as well as a fragment of the knowledge base, for example, with the group criteria $K_{I}$ and the resulting term-evaluation L, can be given as shown in Table 3.

Because the expert puts each variable of $T_{gij}$ authenticity and their reasoning $\mu (T_{gij})$ in the interval [0, 1], then the linguistic variables can be represented in the form of triangular membership functions as done in Reference [44]. This means that each linguistic variable T can be replaced by the neighbor $T^{*}$ with certainty $\mu (T^{*})=1-\mu (T)$. This gives the opportunity to polarize the risks within a group of criteria in order to obtain the resulting term-evaluation according to the knowledge base.

The aggregated score certainty $\mu (T_{gj})$ is calculated according to the following formula [25]:

(2) $$\mu (T_{gj})=\frac{1}{k}\sum_{i=1}^{m}\mu (T_{gij}),g=\left\{C;M;I;F;S\right\},j=\bar{1,n},$$

where $\mu (T_{gj})$ is the estimation of the certainty of those linguistic variables, which coincides with the resulting term-evaluation for the *i*-th criterion by g group of risk criteria, k is their number, and j is the environmental start-up project considered in air transport.

Thus, in the first stage, we obtain the resulting term-evaluation, based on the membership rules, for each group of risk criteria, the considered environmental start-up of the air transport project, and an aggregate assessment of its reliability.

In the second stage of problem solution, the approach described below is used to determine the generalized risk assessment environmental start-up project in air transport for each group of criteria g, to achieve an aggregated risk assessment, as well as its linguistic interpretation.

Next, consider the following mathematical model [25]:

(3) $$R=V\left(x(T_{gj});\mu (T_{gj});O_{gj};O_{R}(S_{j})\right),$$

where $x(T_{gj})$ is the value of a function equal to the numerical interpretation of the resulting term-estimates, $T=\left\{\begin{array}{ccccc} L; & BA; & A; & AA; & H \end{array}\right\}$, $\mu (T_{gj})$ is the aggregated assessment of the certainty of the expert’s thoughts, $O_{gj}$ is a project risk assessment for each group of criteria g, $O_{R}(S_{j})$ is the aggregated risk assessment for environmental start-up projects in the air transport sector across all groups of criteria g, and R is its output linguistic interpretation. V is the operator that matches the output variable R for input variables $x(T_{gj});\mu (T_{gj});O_{gj};O_{R}(S_{j}),j=\bar{1,n}$.

Because the resulting term-assessment $T_{gj}$ has a level of risk content, then its terms can be adequately determined on a percentage scale (0–100%), each of which sets values from interval [a;b], for example L [0, 15], BA [15, 30], A [30, 50], AA [50, 80], and H [80, 100]. That is, a value of 85% risk is treated as “high risk”.

We then consider the dependence of the resulting term evaluation $T_{gj}$ and its certainty $\mu (T_{gj})$ in the form of the *S*-shaped membership function as in References [4, 25], which, in our view, appropriately expresses this dependency.

(4) $$\mu (T_{gj})=\{\begin{array}{cc} 0, & x_{gj}\leq a;\\ 2{\left(\frac{x_{gj}-a}{b-a}\right)}^{2}, & a<x_{gj}\leq \frac{a+b}{2};\\ 1-2{\left(\frac{b-x_{gj}}{b-a}\right)}^{2}, & \frac{a+b}{2}<x_{gj}<b;\\ 1, & x_{gj}\geq b. \end{array}g=\left\{C;M;I;F;S\right\},j=\bar{1,n}.$$

Since the membership function values (aggregated estimation of certainty) and the intervals of numeric values for T are known, then, for each group of criteria g, $x_{gj}$ is expressed from Equation (4) [4].

(5) $$x_{gj}=\{\begin{array}{cc} \sqrt{\frac{\mu (T_{gj})}{2}}(b-a)+a, & 0\leq \mu (T_{gj})\leq 0.5;\\ b-\sqrt{\frac{1-\mu (T_{gj})}{2}}(b-a), & 0.5<\mu (T_{gj})\leq 1. \end{array}$$

Equation (5) denotes that a higher value $x_{gj}$ signifies a greater risk of a project start-up in the appropriate group of criteria. 

For generalized risk assessments of environmental start-up projects in the air transport sector by groups of criteria *g*, the normalized values $x_{gj}$ are obtained, changing the orientation of objectives.

(6) $$O_{gj}=(b-x_{gj})/b,j=\bar{1,n}.$$

The estimates $O_{gj}$,$j=\bar{1,n}$ are normalized and represent a criterion for each group g aggregated risk assessment of the considered environmental start-ups of air transport projects in relation to the resulting thermal ratings and their reliability.

For DMs for each group of criteria, the weight coefficients are denoted as $\left\{p_{C},p_{M},p_{I},p_{F},p_{S}\right\}$ from some interval. Then, the corresponding weighted coefficients are set accordingly.

(7) $$\alpha_{g}=\frac{p_{g}}{\sum_{g}p_{g}},g=\{C;M;I;F;S\},\sum_{g}p_{g}=1.$$

Since all the estimates obtained are normalized by the interval [0, 1], then, in order to obtain a final assessment of the risk of financing the environmental start-up of projects in the air transport sector, the approach below can be used. Depending on the size of the investment, DMs can choose one of the following convolutions:

(8) $$O_{R1}(S_{j})=\frac{1}{\sum_{g}\frac{\alpha_{g}}{O_{gj}}};j=\bar{1,n}—\mathrm{Pessimistic};$$

(9) $$O_{R2}(S_{j})=\prod_{g}{\left(O_{gj}\right)}^{\alpha_{g}};j=\bar{1,n}—\mathrm{Cautious};$$

(10) $$O_{R3}(S_{j})=\sum_{g}\alpha_{g}O_{gj};j=\bar{1,n}—\mathrm{Average};$$

(11) $$O_{R4}(S_{j})=\sqrt{\sum_{g}\alpha_{g}{\left(O_{gj}\right)}^{2}};j=\bar{1,n}—\mathrm{Optimistic}.$$

The resulting estimates $O_{R}(S_{j})$ are normalized and then matched to the output variable R to provide the following scale:

- $r_{1}$ = “Insignificant risk of financing the environmental start-up of the air transport project”;
- $r_{2}$ = “Low risk of financing the environmental start-up of the air transport project”;
- $r_{3}$ = “Average risk of financing the environmental start-up of the air transport project”;
- $r_{4}$ = “High risk of financing the environmental start-up of the air transport project”;
- $r_{5}$= “Critical risk of financing the environmental start-up of the air transport project”.

The linguistic interpretation of the aggregated risk assessment for financing the environmental start-up projects in the air transport sector $R=\{r_{1},r_{2},r_{3},r_{4},r_{5}\}$ is as follows: $O_{R}\in$(0.85, 1]—$r_{1}$; $O_{R}\in$(0.67, 0.85]—$r_{2}$; $O_{R}\in$(0.36, 0.67]—$r_{3}$; $O_{R}\in$(0.21, 0.36]—$r_{4}$; $O_{R}\in$[0, 0.21]—$r_{5}$.

The suggested decision levels are experimentally obtained, and the decision-maker can change them. To improve the accuracy of boundary estimation, one can change the experience of experts in evaluating the environmental start-up projects. Also, depending on the investment opportunities of investors, if necessary, the level of decision-making can also change [45]. 

### 3.4. Generalized Algorithm for Obtaining an Aggregated Risk Assessment for Environmental Start-Up Projects in the Air Transport Sector

Based on the above fuzzy risk assessment model, the environmental start-up of air transport projects can be written as a generalized aggregated estimation algorithm.

**Step 1.** Determine the resulting term-evaluation. 

Based on the data entered, the projects introduced from the start-up, and the built knowledge base, the resulting term-evaluation by Equation (1) for the groups of criteria is determined: KC; KM; KI; KF; KS.

**Step 2.** Determine the aggregated estimation of the reliability of the expert’s thoughts.

The aggregated evaluation certainty $\mu (T_{gj})$,g={C;M;I;F;S},$j=\bar{1,n}$ is calculated according to Equation (2).

**Step 3.** Obtain a generalized risk assessment for projects by groups of criteria g.

For each group of criteria g, calculate the level of risk $x_{gj}$, relative to the percentage scale [a;b] and the resulting term-evaluation $T_{gj}$, using Equation (5). The generalized evaluation $O_{gj}$ risk start-up projects for each group of criteria g is given by Equation (6). 

**Step 4.** Weight coefficients introduced by groups of risk criteria. 

For each group of criteria, DMs set weight coefficients $\left\{p_{C},p_{M},p_{I},p_{F},p_{S}\right\}$ after which, according to Equation (7), the normalized weight coefficients are calculated.

**Step 5.** Aggregated risk assessment calculated for all groups of criteria.

We determine the aggregated risk assessment using one of the convolutions in Equations (8)–(11).

The equate assessment $O_{R}(S_{j})$ with the output variable R is to obtain a linguistic interpretation of the level of risk financing environmental start-up projects in air transport. 

In this way, a fuzzy mathematical model was constructed to obtain an aggregated risk assessment for environmental start-up projects in the air transport sector. The model used expert’s knowledge and reasoning to evaluate the various risk criteria and, based on this, there was an aggregation of views according to the groups of criteria in the final evaluation. 

## 4. Results

The results of the research were tested for an example of the risk assessment of environmental start-up projects in air transport. To simulate the situation, there were three environmental start-up projects $S_{1},S_{2},S_{3}$ (taken from the University Science Park TECHNICOM ecosystem in Kosice and the start-up incubator at the Uzhhorod National University), for which the risk of their financing during the expansion needed to be assessed. The input data for the expert evaluation of start-ups on the proposed set of criteria are listed in Table 4.

The risks of financing start-up projects were evaluated based on the proposed generalized algorithm.

**Step 1**. Determine the resulting term-evaluation. 

Based on the projects introduced from the start-up and the built knowledge base, the resulting term-evaluation was determined.

**Step 2.** Determine the aggregated estimation of the reliability of the expert’s thoughts using Equation (2). 

The results of the calculation of steps 1and 2 are given in Table 5.

**Step 3.** Obtain a generalized risk assessment for projects by groups of criteria g.

For each group of criteria, $x_{gj}$ was calculated using Equation (5) and the generalized assessment $O_{gj}$ risk using Equation (6). For example, to illustrate this, for a group of criteria KC related to the start-up $S_{1}:$ $x_{C1}=30-\sqrt{\frac{1-0.63}{2}}(30-15)=23.55$; $O_{C1}=(100-23.55)/100=0.7645$. The results of all calculations are listed in Table 6. 

**Step 4**. Weight coefficients are set for groups of risk criteria. 

For each group of criteria, DMs set weight coefficients {9; 7; 8; 6; 9} and the normalized weighting coefficients were calculated using Equation (7): {0.23; 0.18; 0.2; 0.16; 0.23}. 

By matching the received assessments $O_{R}$ with the output variable R, the following result was obtained: $S_{1}$—“average risk of financing environmental start-up of the air transport project”; $S_{2}$—“insignificant risk of financing environmental start-up of the air transport project”; $S_{3}$—“low risk of financing environmental start-up of the air transport project”.

From these estimates, the following conclusion could be drawn: the least risky environmental start-up project in the air transport sector, for its financing at the expansion stage, was the project designated as $S_{2}$, with an assessment of 0.8518 and insignificant financing risk. The fuzzy model developed enhanced the accuracy and objectivity of the assessment, as it used linguistic risk assessments on the one hand and, on the other, the expertise and competencies of experts in the form of a value of “certainty” of their considerations for different risk criteria. On this basis, opinions were aggregated by groups of criteria into a final evaluation. Quantitative assessment increases the validity of decision-making, and, on this basis, the decision-maker can compare projects and select qualitative ones for financing.

## 5. Discussion

The fuzzy model of risk assessment for environmental start-up projects in the air transport sector, as part of this research, will increase the degree of validity of decision-making regarding the financing of these investors by the investing public at the stage of market expansion. The model is based on expert knowledge, uses the linguistic variables, reveals the fuzzy input estimates, raises the objectivity of expert judgments, and combines experts’ opinions in the benchmark groups of criteria for the final assessment of the risk of environmental start-ups in the air transport sector.

The developed fuzzy risk assessment model for financing the start-up projects in the air transport sector has several advantages, such as the following

- increasing the objectivity of expert assessments in project risk assessment using inbound linguistic variables and the credibility of expert estimates, where their mission and developed knowledge base do not depend on the number of criteria in the groups;
- they can be increased if needed, which also changes the level of decision-making;
- the model combines the criteria group’s views in the final risk assessment of the environmental start-up project and derives linguistic interpretation.

The disadvantages of this model include the use of different types of membership functions (triangular for the linguistic variable and its authenticity of an assignment, as well as for the dependence on the resulting evaluation term), which can lead to ambiguity of results.

Rationalizing the risk assessment taken to finance environmental start-up projects in air transport brings the benefits of the developed model. The reliability of the results is achieved by the proper use of the apparatus of fuzzy sets, which is confirmed by the results of the research. 

Therefore, the Slovak Republic must seek intensive solutions to the start-up ecosystem’s penetration into the European start-up system. It creates various support schemes, mechanisms, and initiatives. In the Slovak Republic, there is no commercial exploitation of applied research and creation of so-called spin-offs. Although, in 2015, Slovakia adopted the measure of support for the old world and the old world eco-system, according to many experts, the entry of the state into its regulatory measures and instruments (working groups, commissions, ratings, etc.) created space for customers, overpriced electronic services, corruption, and inefficient use of public resources. It is, therefore, important to develop and support initiatives where the essence of a start-up as a platform for business democratization and corporate social responsibility is undisturbed.

The study looked for links between macroeconomic characteristics—the critical growth points of start-ups and their impacts—expressed through the values of economic indicators, which indicate the state and development of the old system in Slovakia. Despite the support of government structures, the development of start-ups is also dependent on other entities in the start-up ecosystem, as well as changes in their internal environment, which need to be monitored and evaluated continuously. Macroeconomic monitoring mechanisms are not sufficient for this, but it is important to monitor and evaluate this environment by developing models and using evaluation methodologies to minimize the risk of start-up survival. Our analytical study shows the significant potential of fuzzy models for assessing the risks of environmental start-up projects in a selected aviation sector. This exploration develops relatively slowly due to its specificity, methodologies, and data requirements. The presentation of the fuzzy model and the potential for subsequent software solutions will provide valuable ideas for the development of the methodological platform in its horizontal and vertical lines. In the horizontal line, we see a space for developing models differentiated by a sector, where there may be partial modification of the evaluation areas (characteristics, constructions). In the vertical line, we see space for developing models that reflect risk areas at different stages of start-up development, which can eliminate extinction or poor performance in other stages of development. It also highlights the role of expert teams whose professionalism, knowledge, and experience can help discover other aspects of the survival and successful development of start-ups. We intend to create databases with criteria that influence the decision-making processes of investors, as well as other stakeholders of the start-up-ecosystem.

Each model operates with some limitations; the platform of fuzzy models and their associated methodologies also provide room for complementary application of several types of structural analysis, which, in their initial phase, could determine the impact and dependence of variables on various processes and financial aspects of start-ups. These should be explored depending on the specific type of start-ups. This is a challenging research area due to the strong individuality of the start-up projects and their high degree of innovation.

The results of our study will also support the development of economic indicators for assessing the potential success rate of start-up projects and, consequently, for national and international benchmarking. Our follow-up research area will also examine the forecasted development of the potential success of start-up projects linked to the country’s macroeconomic indicators, as well as their economic and innovation performance. As some studies indicated [9], many important indicators are missing in the available EU macroeconomic databases, and those that are often monitored are incomplete (data on patents and inventions in countries, etc.) The results of our study will provide valuable information to experts dealing with regional strategic concepts, regional innovation, and economic development plans, as well as the financial sector, and will support the development of new forms of financing for this future segment of SMEs.

## 6. Conclusions

Start-ups are key drivers of the structural changes required for the economy and economic growth and for maintaining their innovative performance and competitiveness. They ensure the development of innovative products and services that will create new markets, or redefine and expand existing ones, creating a strong growth potential. Start-ups as the determinants of structural changes can change the way in which companies, sectors, and the public sector operate. The attention of the Government of the Slovak Republic is on developing the Slovak start-up system, while linking the education and research system with the business environment remains an important issue.

Investigation of the actual task involved the risk assessment for environmental start-up projects in air transport sector, at the stage of gaining a successful start-up project. The result was an output quantitative assessment which increases the validity of decision-making, and, on its basis, the decision-maker can compare projects and select qualitative ones for financing. At the same time, the following results were obtained for the first time:

- The set of 21 criteria, for assessing the risk of developing environmental start-up projects in the air transport sector, was divided into five groups that revealed different aspects of risk assessment at the project extension stage. The inputs were presented in the form of a linguistic risk assessment, a set of five linguistic variables, and a number of expert opinions.
- The rules for membership in the resulting term evaluation for risk criteria groups were set out to build the knowledge base, where the level of decision-making can be changed, which does not depend on the number of criteria per group.
- The model of fuzzy risk assessment for environmental start-up projects in the air transport sector was developed, based on expert knowledge, using linguistic variables, and it reveals the uncertainty of input data, as well as integrates experts’ opinions into groups of criteria in the final assessment of risk with linguistic interpretation.
- A generalized five-step algorithm for obtaining an aggregated risk assessment for environmental start-up projects in air transport sector was constructed.
- The developed fuzzy model was tested in a risk assessment example to finance three environmental start-ups of air transport projects at the stage of business expansion.

The future work of researchers will focus on three dimensions. The first is to create an SW tool for the risk assessment of environmental start-up projects within the pre-investment phase of the project life cycle based on the presented expert fuzzy model in the paper. The creation of software based on the risk assessment model developed to finance environmental start-up projects in the air transport sector will be a useful tool to support the decision support systems of investment institutions (venture funds, business angels, and crowdfunding investment platforms) in financing the environmental start-up projects at the market conquest stage. The second is to develop a new expert model for the risk assessment of aviation environmental project implementation as a part of the investment life cycle of projects. The third is to develop the SW tool for the risk assessment of aviation environmental project implementation as a part of the investment life cycle of projects (such as the web application or fixed application). 

The issue of the impact of aviation on our public health under the United Nations Sustainable Development Agenda (2030), which supports the spirit of sustainable development objectives, which also aims for climate action under Objective 13, requires effective solutions. Proposals for environmental projects in the aviation sector are an important source of innovation. Therefore, the authors examined new tools and a methodology for their assessment with emphasis on risk assessment in accordance with the aim of this paper.

## Figures and Tables

**Table 1:** Input data.

The Name of the Criteria	$S_{1}$		$S_{2}$		…	$S_{n}$	
$K_{g1}$	$T_{g11}$	$\mu (T_{g11})$	$T_{g12}$	$\mu (T_{g12})$	…	$T_{g1n}$	$\mu (T_{g1n})$
$K_{g2}$	$T_{g21}$	$\mu (T_{g21})$	$T_{g22}$	$\mu (T_{g22})$	…	$T_{g2n}$	$\mu (T_{g2n})$
$K_{gm}$	$T_{gm1}$	$\mu (T_{gm1})$	$T_{gm2}$	$\mu (T_{gm2})$	…	$T_{gmn}$	$\mu (T_{gmn})$

**Table 2:** Definition of the criteria and their groups.

Criteria Groups	Label of Criteria	Definition of Criteria
KC	$KC_{1}$	The risk of losing a large client (the absence of signed contracts with aviation enterprises or companies operating in the air transport industry);
	$KC_{2}$	Risk of losing the supplier of raw materials (replacing the supplier is always accompanied by new risks arising from the new relationship);
	$KC_{3}$	The risk of losing market share (the market is likely to acquire new environmental start-ups of air transport, which is likely to take away customers);
	$KC_{4}$	The risk of unsecured resources (this risk is linked to inappropriate formation of resource stocks, particularly the expansion of production).
KM	$KM_{1}$	The risk of lowering the level of management (when the leaders of the start-up team act in their own interest, forgetting the initial arrangements among investors);
	$KM_{2}$	The risk of lowering the quality of the processes in the start-up team (mainly due to the loss of motivation of the team members, which directly affects the quality of the work);
	$KM_{3}$	The risk of reducing productivity of the start-up team (occurs when there is a crisis in the system of motivation);
	$KM_{4}$	Personnel risks (aspects related to lack of skilled workers, violations of labor, and executive discipline).
KI	$KI_{1}$	The risk of inefficiency of investment (when the investment cost is higher than the return on investment);
	$KI_{2}$	Risk of failing to achieve return on investment capital (failure to reach projected return on project start-up);
	$KI_{3}$	The risk of disrupting the timing of the creation of production assets (delay in commissioning production assets— a typical violation of project investment plans);
	$KI_{4}$	The risk of exceeding the amount of investment costs (a characteristic defect of the financial plan and of the part responsible for calculating the investment costs, usually due to lack of detail in business planning);
	$KI_{5}$	The risk of a lack of investment capital (closely linked to the previous risk and accompanied by a threat to the cost of financing the project).
KF	$KF_{1}$	Risk of loss (arises in relation to price changes when sudden expenses cover revenue);
	$KF_{2}$	The risk of losing of solvency (perhaps a large-scale payment, which was not considered and, therefore, was not prepared for, or when there is a force majeure need for large-scale payments).
	$KF_{3}$	The risk of a suboptimal capital price (when it results in higher financial cost than operating profit).
KS	$KS_{1}$	The risk of ineffective new innovative investments (when the investment cost is higher than the return on innovation performance);
	$KS_{2}$	The risk of ineffective new innovative ideas (innovative upgrading of environmental start-up projects must focus on increasing sales trend);
	$KS_{3}$	Risks of violating the conditions of development of environmental start-up projects (the period of implementation of innovations is measured in months and weeks, where a delay means losing market);
	$KS_{4}$	Risks of technological environmental start-up projects (the risk relates to the technology of organizational change, when insufficient attention paid to the transition to the stages of change resulted in the failure of implementation);
	$KS_{5}$	Risk of resource scarcity when designing environmental start-up projects (sometimes the difficulty of accessing scarce resources by these specialists may be considered specialized, as well as technologies and components whose access is limited) is overlooked.

**Table 3:** Fragment of knowledge base.

№ Rules	$K_{I1}$	$K_{I2}$	$K_{I3}$	$K_{I4}$	$K_{I5}$	Resulting Term Evaluation
1	L^1^	L	L	L	BA^2^	L
2	L	L	L	BA	BA	
3	L	L	L	BA	L	
4	L	L	BA	BA	L	
5	L	L	BA	L	L	
6	L	BA	BA	L	L	
…	…	…	…	…	…	

^1^ “low risk”, ^2^ “risk below average”.

**Table 4:** Input expert evaluation risk of start-up projects.

Criteria Groups	The Name of the Criteria	$S_{1}$		$S_{2}$		$S_{3}$	
		*T*	μ(T)	*T*	μ(T)	*T*	μ(T)
KC	$KC_{1}$	L^1^	0.6	L	0.9	BA^2^	0.8
	$KC_{2}$	BA	0.7	L	0.8	BA	0.6
	$KC_{3}$	BA	0.8	L	0.7	L	0.4
	$KC_{4}$	A^3^	0.6	BA	0.9	L	0.8
KM	$KM_{1}$	A	0.6	L	0.8	BA	0.6
	$KM_{2}$	A	0.9	A	0.4	BA	0.8
	$KM_{3}$	A	0.8	L	0.7	A	0.8
	$KM_{4}$	BA	0.7	BA	0.8	BA	0.8
	$KI_{1}$	AA^4^	0.9	L	0.8	BA	0.7
	$KI_{2}$	A	0.7	L	0.8	A	0.6
KI	$KI_{3}$		0.6	BA	0.8	A	0.7
	$KI_{4}$	AA	0.9	L	0,7	L	0.9
	$KI_{5}$	L	0.6	BA	0.6	BA	0.6
	$KF_{1}$	A	0.8	L	0.7	BA	0.7
KF	$KF_{2}$	AA	0.7	BA	0.6	BA	0.6
	$KF_{3}$	AA	0.6	L	0.8	BA	0.8
KS	$KS_{1}$	BA	0.8	BA	0.6	A	0.5
	$KS_{2}$	A	0.9	BA	0.8	BA	0.6
	$KS_{3}$	A	0.8	BA	0.8	A	0.8
	$KS_{4}$	BA	0.7	BA	0.7	A	0.8
	$KS_{5}$	A	0.6	L	0.8	BA	0.8

^1^ “low risk”, ^2^ “risk below average”, ^3^ “average risk”, ^4^ “risk above average”.

**Table 5:** The results and aggregated estimates of expert confidence.

Criteria Groups	$S_{1}$		$S_{2}$		$S_{3}$	
	*T*	μ(T)	*T*	μ(T)	*T*	μ(T)
KC	BA	0.63	L	0.8	L	0.53
KM	A	0.77	BA	0.43	BA	0.73
KI	A	0.33	L	0.77	BA	0.5
KF	AA	0.65	L	0.75	BA	0.77
KS	BA	0.63	BA	0.73	BA	0.63

**Table 6:** The generalized ratings.

Groups of Criteria	$S_{1}$		$S_{2}$		$S_{3}$	
	*x*	*O*	*x*	*O*	*x*	*O*
KC	23.55	0.7645	10.26	0.8974	7.73	0.9227
KM	43.22	0.5678	18.26	0.8174	24.5	0.755
KI	38.12	0.6188	9.91	0.9009	22.5	0.775
KF	67.45	0.3255	9.7	0.903	24.19	0.7581
KS	23.55	0.7645	24.5	0.755	23.64	0.7636
