# Correction: A Fuzzy Model of Risk Assessment for Environmental Start-Up Projects in the Air Transport Sector. *Int. J. Environ. Res. Public Health* 2019, *16*, 3573

**DOI:** 10.3390/ijerph16234850

**Published:** 2019-12-02

**Authors:** Volodymyr Polishchuk, Miroslav Kelemen, Beáta Gavurová, Costas Varotsos, Rudolf Andoga, Martin Gera, John Christodoulakis, Radovan Soušek, Jaroslaw Kozuba, Peter Blišťan, Stanislav Szabo

**Affiliations:** 1 Faculty of Information Technologies, Uzhhorod National University, 88000 Uzhhorod, Ukraine; 2 Faculty of Aeronautics, Technical University of Kosice, 04121 Kosice, Slovakia; miroslav.kelemen@tuke.sk (M.K.); rudolf.andoga@tuke.sk (R.A.); stanislav.szabo.2@tuke.sk (S.S.J.); 3 Research and Innovation Centre Bioinformatics, USP TECHNICOM, Technical University of Košice, 040 01 Kosice, Slovakia; beata.gavurova@tuke.sk; 4 Department of Physics, National & Kapodistrian University of Athens, GR-15784 Athens, Greece; covar@phys.uoa.gr (C.V.); ichristo@phys.uoa.gr (J.C.); 5 Faculty of Mathematics, Physics and Informatics, Comenius University, Bratislava, Mlynska dolina 84248, Slovakia; mgera@fmph.uniba.sk; 6 Faculty of Transport Engineering, University of Pardubice, 53210 Pardubice, Czech Republic; radovan.sousek@upce.cz; 7 Faculty of Transport, Silesian University of Technology, 44100 Gliwice, Poland; jaroslaw.kozuba@polsl.pl; 8 Faculty of Mining, Ecology, Process Control and Geotechnology of Aeronautics, Technical University of Kosice, 04121 Kosice, Slovakia; peter.blistan@tuke.sk

The authors wish to make the following correction to their paper [1]. Mr. Jakub Hospodka has requested that he be removed from the list of contributing authors as he collaborated only as the authors’ project partner. The authors, therefore, wish to replace:

Volodymyr Polishchuk ^1,^*, Miroslav Kelemen ^2^, Beáta Gavurová ^3^, Costas Varotsos ^4^, Rudolf Andoga ^2^, Martin Gera ^5^, John Christodoulakis ^4^, Radovan Soušek ^6^, Jaroslaw Kozuba ^7^, Jakub Hospodka ^8^, Peter Blišťan ^9^ and Stanislav Szabo Jr. ^2^

^1^  Faculty of Information Technologies, Uzhhorod National University, Uzhhorod 88000, Ukraine; volodymyr.polishchuk@uzhnu.edu.ua
^2^  Faculty of Aeronautics, Technical University of Kosice, Kosice 04121, Slovak Republic; miroslav.kelemen@tuke.sk (M.K.); rudolf.andoga@tuke.sk (R.A.); stanislav.szabo.2@tuke.sk (S.S.j)
^3^  Research and Innovation Centre Bioinformatics, USP TECHNICOM, Technical University of Košice, 040 01 Kosice, Slovak Republic; beata.gavurova@tuke.sk
^4^  Department of Physics, National & Kapodistrian University of Athens, Athens GR-15784, Greece; covar@phys.uoa.gr (C.V.); ichristo@phys.uoa.gr (J.C.)
^5^  Faculty of Mathematics, Physics and Informatics, Comenius University in Bratislava, Bratislava, Mlynska Dolina 84248; Slovak Republic; mgera@fmph.uniba.sk
^6^  Faculty of Transport Engineering, University of Pardubice, Pardubice 53210, Czech Republic; radovan.sousek@upce.cz
^7^  Faculty of Transport, Silesian University of Technology, Gliwice 44100, Poland; jaroslaw.kozuba@polsl.pl
^8^  Faculty of Transport, Czech Technical University in Praque, Praque 16000, Czech Republic; xhospodka@fd.cvut.cz
^9^  Faculty of Mining, Ecology, Process Control and Geotechnology of Aeronautics, Technical University of Kosice, Kosice 04121, Slovak Republic; peter.blistan@tuke.sk

with:

Volodymyr Polishchuk ^1,^*, Miroslav Kelemen ^2^, Beáta Gavurová ^3^, Costas Varotsos ^4^, Rudolf Andoga ^2^, Martin Gera ^5^, John Christodoulakis ^4^, Radovan Soušek ^6^, Jaroslaw Kozuba ^7^, Peter Blišťan ^8^ and Stanislav Szabo, Jr. ^2^

^1^  Faculty of Information Technologies, Uzhhorod National University, 88000 Uzhhorod, Ukraine
^2^  Faculty of Aeronautics, Technical University of Kosice, 04121 Kosice, Slovakia; miroslav.kelemen@tuke.sk (M.K.); rudolf.andoga@tuke.sk (R.A.); stanislav.szabo.2@tuke.sk (S.S.J.)
^3^  Research and Innovation Centre Bioinformatics, USP TECHNICOM, Technical University of Košice, 040 01 Kosice, Slovakia; beata.gavurova@tuke.sk
^4^  Department of Physics, National & Kapodistrian University of Athens, GR-15784 Athens, Greece; covar@phys.uoa.gr (C.V.); ichristo@phys.uoa.gr (J.C.)
^5^  Faculty of Mathematics, Physics and Informatics, Comenius University, Bratislava, Mlynska dolina 84248, Slovakia; mgera@fmph.uniba.sk
^6^  Faculty of Transport Engineering, University of Pardubice, 53210 Pardubice, Czech Republic; radovan.sousek@upce.cz
^7^  Faculty of Transport, Silesian University of Technology, 44100 Gliwice, Poland; jaroslaw.kozuba@polsl.pl
^8^  Faculty of Mining, Ecology, Process Control and Geotechnology of Aeronautics, Technical University of Kosice, 04121 Kosice, Slovakia; peter.blistan@tuke.sk

The authors would like to apologize for any inconvenience caused to the readers by the change. The change does not affect the scientific results. The manuscript will be updated, and the original will remain online on the article webpage, with a reference to this correction.
